# Meta-Analysis of Adiponectin as a Biomarker for the Detection of Metabolic Syndrome

**DOI:** 10.3389/fphys.2018.01238

**Published:** 2018-09-19

**Authors:** Zhengtao Liu, Shuheng Liang, Shuping Que, Lin Zhou, Shusen Zheng, Adil Mardinoglu

**Affiliations:** ^1^Key Laboratory of Combined Multi-Organ Transplantation, Ministry of Public Health and Key Laboratory of Organ Transplantation of Zhejiang Province, Hangzhou, China; ^2^Division of Hepatobiliary and Pancreatic Surgery, Department of Surgery, First Affiliated Hospital, School of Medicine, Zhejiang University, Hangzhou, China; ^3^Department of Pediatrics, Women and Children's Hospital of Guangxi, Nanning, China; ^4^Science for Life Laboratory, KTH-Royal Institute of Technology, Stockholm, Sweden; ^5^Department of Biology and Biological Engineering, Chalmers University of Technology, Gothenburg, Sweden; ^6^Centre for Host–Microbiome Interactions, Dental Institute, King's College London, London, United Kingdom

**Keywords:** adiponectin, metabolic syndrome, diagnostic accuracy, prediction, meta-analysis

## Abstract

Previous studies revealed the potential significance of circulating adiponectin levels with respect to the diagnosis and prediction of metabolic syndrome, but uncertainty has been noted across different cohorts. Systematic evaluation was performed for diagnostic accuracy and predictivity of adiponectin variation for metabolic syndrome in enrolled studies including 1,248 and 6,020 subjects, respectively. Adiponectin can identify metabolic syndrome with moderate accuracy (area under the curve = 0.81, 95% CI: 0.77–0.84). Heterogeneity analysis revealed that an increasing index of insulin resistance was independently associated with improving the performance of adiponectin upon metabolic syndrome diagnosis (ratio of diagnostic odds ratio = 3.89, 95% CI: 1.13–13.9). In addition, reductions in adiponectin were associated with increasing metabolic syndrome incidence in a linear dose-response manner. The risk of hypoadiponectinemia with metabolic syndrome was especially increased in men (*P* < 0.05). Further Mendelian randomization analysis identified that the amplified risk could be attributed to increased susceptibility (up to 7%) to insulin resistance compared with women. In conclusion, adiponectin measurement might have potential benefits in the detection of metabolic syndrome. Factors that affect insulin resistance should be considered for adjustment in future assessments.

## Introduction

Metabolic syndrome (MetS) is defined as a panel of endocrine disorders, including central adiposity, dyslipidemia, hyperglycemia and hypertension (Kaur, 2014). Cored by insulin resistance, MetS (diagnosed using different criteria) is a crucial mediator in promoting the incidence of non-alcoholic fatty liver disease (NAFLD), type 2 diabetes, cardiovascular disease (CVD) and subsequent mortality (Ford, 2005; Gami et al., 2007). Given the increasing health burden from excessive calorie intake and absence of physical inactivity, MetS is a pandemic disease that affects greater than 25% of the global population and has caused comprehensive concerns worldwide (Grundy, 2008, 2016). MetS development involves numerous cytokines (Matsuzawa, 2006; Rizvi, 2009; Maury and Brichard, 2010), and systematic knowledge of key factors is beneficial for further mechanistic investigation and disease prevention.

Adiponectin (ADPQ), which is encoded by the AdipoQ gene located on chromosome 3q27.3, is a 244-amino acid pleiotropic hormone with a molecular weight of approximately 30 kDa for its basic subunit. ADPQ is exclusively secreted by adipocytes, and abundant levels in the circulation are eliminated by liver. ADPQ consists of different multimerizations, including low molecular weight (LMW) and high molecular weight (HMW) hexamers. The HMW multimer is considered the major active functional component of ADPQ (Robinson et al., 2011). Previous studies revealed that adiponectin played a causal role in maintaining energy homeostasis via regulation of glucose and lipid metabolism. Particularly, adiponectin can alleviate insulin resistance by stimulating the cellular glucose uptake and organ/circulating fatty acid oxidation via activation of key proteins, including adenosine monophosphate-activated protein kinase (AMPK) and peroxisome proliferator activated receptor gamma (PPAR-γ) (Yamauchi et al., 2001, 2002; Tschritter et al., 2003; Kadowaki et al., 2006). ADPQ is an effective biomarker for the prediction and risk classification of insulin resistance (IR) and its related complications (Tschritter et al., 2003; Yamamoto et al., 2004; Hara et al., 2006). Furthermore, pre-clinical studies in mice demonstrated the therapeutic potential for ADPQ administration on improving insulin sensitivity. ADPQ is considered an ideal target for drug development to treat IR-related metabolic disorders (Yamauchi et al., 2001; Shetty et al., 2009).

MetS involves systemic insulin resistance with a collection of risk indicators on cardiovascular disease (Roberts et al., 2013). Genetic studies also identified that hypoadiponectinemia shared common genetic constitutions with MetS pathogenesis (Comuzzie et al., 2001; Yang and Chuang, 2006). Hence, the close relationship between ADPQ and MetS risk is not surprising. Even in obese children, ADPQ also exhibited a decreasing tendency in subjects with insulin resistance and potentially influenced subsequent MetS development (Weiss et al., 2004).

Epidemiology surveys provide clues for mechanistic study and approaches to estimate its diagnostic potential. Accordingly, several longitude studies identified that circulating ADPQ might predict MetS incidence even after adjusting for crucial metabolic confounders, such as obesity (Seino et al., 2009; Juonala et al., 2011; Nakashima et al., 2011; Kim et al., 2013a; Hata et al., 2015; Lindberg et al., 2017). Otherwise, ADPQ was also measured to evaluate its accuracy upon MetS diagnosis across studies with populations from different ethnicities and age groups (Ogawa et al., 2005; Gilardini et al., 2006; Mojiminiyi et al., 2007; Lee et al., 2008; Boyraz et al., 2013; Patel et al., 2015). However, the utility of ADPQ as a candidate biomarker in MetS prediction and risk assessment seems paradoxical across studies with uncertain hazard extents for potential differences regarding ethnic origin, gender, samples, comparisons, obesity or other disease status.

To date, previous evidence-based studies only assessed the impact of ADPQ variation on the development of individual features, such as hypertension and hyperglycemia (Li et al., 2009; Kim et al., 2013b). While impact of ADPQ on MetS as systemic manifestation of metabolic disturbance was not assessed (Eckel et al., 2005). To fill in this gap, it is worthwhile to systematically summarize the adiponectin-MetS relationship to provide comprehensive knowledge to improve the diagnosis and treatment of MetS.

Therefore, our current study mainly analyzed the following parameters: (1) accuracy of circulating ADPQ upon MetS diagnosis; (2) causal impacts of varied blood ADPQ on MetS development based on published results. Factors causing heterogeneity were also assessed by subgroup analysis.

## Methods

This study was conducted strictly according to the Preferred Reporting Items for Systematic Reviews and Meta Analyses (PRISMA) criteria (Moher et al., 2009). Details of reporting items are presented in the Supplementary PRISMA Checklist. The study was divided into two interrelated topics based on the analysis of ADPQ as a potential biomarker in MetS diagnosis and prediction. The first goal was to estimate the diagnostic accuracy of ADPQ as a potential tool for MetS detection (namely, accuracy analysis). The second goal was to assess the relationship between blood ADPQ and MetS risk based on longitudinal studies (namely, risk assessment).

### Search strategy

Three electronic databases, including PubMed (National Library of Medicine, Bethesda, Maryland, USA), Embase (Elsevier, Amsterdam, Netherlands) and ISI Web of Science (Thomson Reuters, London, UK), were searched up to March 1st, 2018 with the help of an experienced librarian. The criterion for the enrolled studies was that they should be performed with humans and published as original full papers without language restriction.

The following subject terms were applied to the literature search: “adiponectin”; “ADIPOQ”; “ADPQ”; “ACDC”; “GBP-28”; “apM1”; “Acrp30”; “metabolic syndrome”; “MetS”; “MS”; “syndrome X” and “insulin resistance syndrome.” More details about the search strategy in a separate database are presented in Table S1.

Additionally, relevant papers were searched manually if omitted in routine retrieval but identified in reference lists of enrolled studies.

### Eligibility criteria

For accuracy analysis, only studies with content that referred to the precision of ADPQ measurement upon MetS diagnosis were considered. In addition, qualified studies should meet the following criteria: 1. necessary data to construct 2 × 2 table for MetS by ADPQ test at best cutoff and largest area under the receiver operating characteristic (AUROC) curves should be provided or extractable by software; 2. MetS was diagnosed with pre-defined criteria.

With regard to risk assessment, we only included prospective studies related to the impact of blood ADPQ on MetS occurrence that simultaneously met the following criteria: (1) no patients with MetS at baseline; (2) pre-defined definition for MetS diagnosis; (3) hazard ratios based on ADPQ variation were provided or could be derived from calculations.

All studies that met the inclusion criteria were enrolled regardless of study population or sample size. If duplicate data were observed in different studies, we only collected the most recent dataset with detailed information.

### Data extraction

Two authors (ZL and SQ) independently extracted information from eligible studies with predesigned unified standardized reporting forms separated by study topics. If relevant information was not provided directly in the enrolled study but presented in attached diagrams, available data would be digitized and extracted via Plot Digitizer software (downloaded on http://plotdigitizer.sourceforge.net/).

For accuracy analysis, the following information was extracted: data source (author, country and publication year), population characteristics (age, gender, number), study design, sampling (ADPQ forms, methods and samples for measurements), definition and prevalence of MetS, and accuracy of ADPQ on MetS diagnosis (sensitivity, specificity, and corresponded AUROC).

Similarly, most items in the accuracy analysis were also included in reported information for risk assessment. However, data accuracy was replaced by MetS risk based on ADPQ variations, including comparison, effect size, calculation and adjusted covariates.

### Rescaling of covariates

The HOMA-IR index was calculated using the following formula when it was not directly provided in the original study: fasting insulin (μU/L) × fasting glucose (nmol/L)/22.5 (Matthews et al., 1985; Whiting et al., 2011). Regarding combinations of quantitative covariates from different subgroups, the data were calculated based on number, mean level and related standard deviation according to pre-defined formulas (Higgins and Green, 2011).

### Quality assessment

Two investigators performed quality assessment for each enrolled study based on different scoring systems divided by research topics.

For accuracy analysis, we adopted the QUADAS-2 scale (Whiting et al., 2011) to evaluate potential heterogeneity and applicability of enrolled studies. The checklist contained four items related to subject's selection: index test, standard reference, study flow and timing (Supplementary QUADAS-2 checklist). Risk of bias for each item in individual study was rated as “low,” “high,” and “unclear” and summarized by proportion. For risk assessment, we chose the NOS checklist designed for nonrandomized cohort studies (Stang, 2010; Wells et al., 2016) to evaluate the potential confounders that caused bias in pooled results. Accordingly, we modified some items to better adapt this scale to our quality assessment (Supplementary NOS checklist). In general, the NOS included nine items from three dimensions, including cohort selection, comparability, and outcome assessment. Studies that received a score greater than 6 were considered high quality.

### Data synthesis and statistical analysis

Pooled test performance of ADPQ detection on MetS diagnosis was evaluated by calculating summary measures of sensitivity (SEN), specificity (SPE), positive likelihood ratio (PLR), negative likelihood ratio (NLR), diagnostic odds ratio (DOR) and their corresponding 95% confidence intervals (CI) within a random-effect framework based on bivariate original data (true positives [TP], false positives[FP], false negatives [FN], and true negatives [TN]) extracted from studies (Reitsma et al., 2005). Negative predictive value (NPV) and positive predictive value (PPV) in cohorts with different MetS prevalence were estimated by pooled SEN and SPE (Altman and Bland, 1994). ROC curves were plotted using a hierarchical regression model (Rutter and Gatsonis, 2001) based on paired sensitivity and specificity from individual studies. Summarized AUROC was calculated to present the test performance, and synthesized diagnostic accuracy was classified as low (0.5–0.7), moderate (0.7–0.9), and high (0.9–1) based on area under curve (AUC) values according to prior criteria (Swets, 1988). The significance of the threshold effect was tested by Spearman correlation between logit SEN/1-SPE (Zamora et al., 2006). The areas under ROC curves across different cohorts were compared by Z test. Diagnostic accuracy was estimated in subgroups categorized by participant features (age, gender), sample size, study design, ADPQ measurement (cutoff, specimen, methods), and MetS definition/prevalence. A multi-covariate meta-regression model was implemented to explore the impact of potential confounders on diagnostic accuracy based on prior subgroup analysis.

For assessment of predictive validity, we extracted multi-covariates adjusted, gender-specific OR values and corresponding 95% CIs (if provided) from original studies to evaluate the risk of hypoadiponectinemia associated with MetS occurrence. Quantitative variation of ADPQ by dichotomous covariate (i.e., gender) was calculated by standardized mean differences (SMD) and corresponding 95% CI. The overall effect was assessed by integration of MetS risk compared between groups with highest and lowest ADPQ levels. Risk extent was transferred by obtaining the reciprocal of given indicator, i.e., 1/OR as proposed before (Tierney et al., 2007), if the control was set as the group with the highest ADPQ values. Cumulative incidence rate of MetS was calculated by the previously mentioned formulation (McDermott et al., 2010). The data were combined based on subject number, mean level and corresponding standard deviance (SD) using a previously described formula (Higgins and Green, 2011). Furthermore, a dose-response meta-analysis of the risk of MetS occurrence associated with a 1 mg/L increase in ADPQ was performed by GLS for trend models (Greenland and Longnecker, 1992; Orsini et al., 2006) based on median ADPQ dose, number of total participants/MetS cases, risk measures and 95% CI compared with subjects with baseline ADPQ values. Then, we assessed the potential non-linearity of the dose-response relationship between ADPQ and MetS risk using two-step GLST methods. Specifically, the slope line was estimated in each individual study using restricted cubic spline functions at four fixed knots assigned with 5th, 35th, 65th, and 95th percentiles of the distribution of the ADPQ level and combined with the overall average spline using the maximum likelihood method in a random-effects model (Harre Jr et al., 1988; Desquilbet and Mariotti, 2010; Orsini et al., 2011). A non-linearity test was implemented based on the null hypothesis that the coefficient of combined spline was equal to 0. Median ADPQ dose that was not directly provided in studies was assigned as the midpoint of the upper and lower limit value in each specific category. For open ended data, the median dose was assigned as lower bounds × 1.2 in the highest group or upper bounds × 0.8 in the lowest group (Berlin et al., 1993).

With regard to the heterogeneity analysis, subgroup analysis categorized by participant features (age, gender, ethnicity, BMI, HOMA-IR), sample size, follow-up and disease definition/prevalence was performed on the pooled dose-response risk of ADPQ variation with MetS incidence. The interaction of potential confounders on ADPQ-MetS association was assessed using a Mendelian randomization approach as previously described (Smith and Ebrahim, 2004). Sensitivity analysis was also conducted to assess the reliability of the effect estimates by re-evaluation of pooled risk on the remaining results after sequentially omitting each individual study.

Publication bias was estimated and presented by different methods. For diagnostic accuracy, publication bias was assessed visually by scatter plot based on log-transformed DOR vs. the inverse of the square root of effective sample sizes (ESS, Deek's funnel plot). And quantitative tests of their associated asymmetry were assessed using a regression model (Deeks et al., 2005). For risk assessment, publication bias was detected using Begg's correlation and Egger's asymmetry test and visualized by Begg's funnel plot (Begg and Mazumdar, 1994; Egger et al., 1997). A measure of statistical heterogeneity across individual studies was quantified using the *I*^2^ test. The threshold for low, moderate and high degree of inconsistency was assigned as 25, 50, and 75%, respectively (Higgins et al., 2003). A random-effect model was applied to estimate the pooled effects with significant heterogeneity (*I*^2^ > 50%), whereas a fixed-effect model was used for results with less heterogeneity (*I*^2^ < 50%), except for specific illustrations (DerSimonian and Laird, 1986).

All calculations were performed by Meta-DiSc (version 1.4) and STATA (Stata/SE 12.0 for Windows, College Station, TX, USA) software. A two-sided *P-*value < 0.05 was considered statistically significant.

## Results

This study was performed and reported strictly according to the statement on Preferred Reporting Items for Systematic Reviews and Meta-Analyses (PRISMA) (Moher et al., 2009). More details are presented in the Supplementary PRISMA Checklist and flow diagram (Figure S1).

### Literature retrieval and study selection

**Figure 1:**
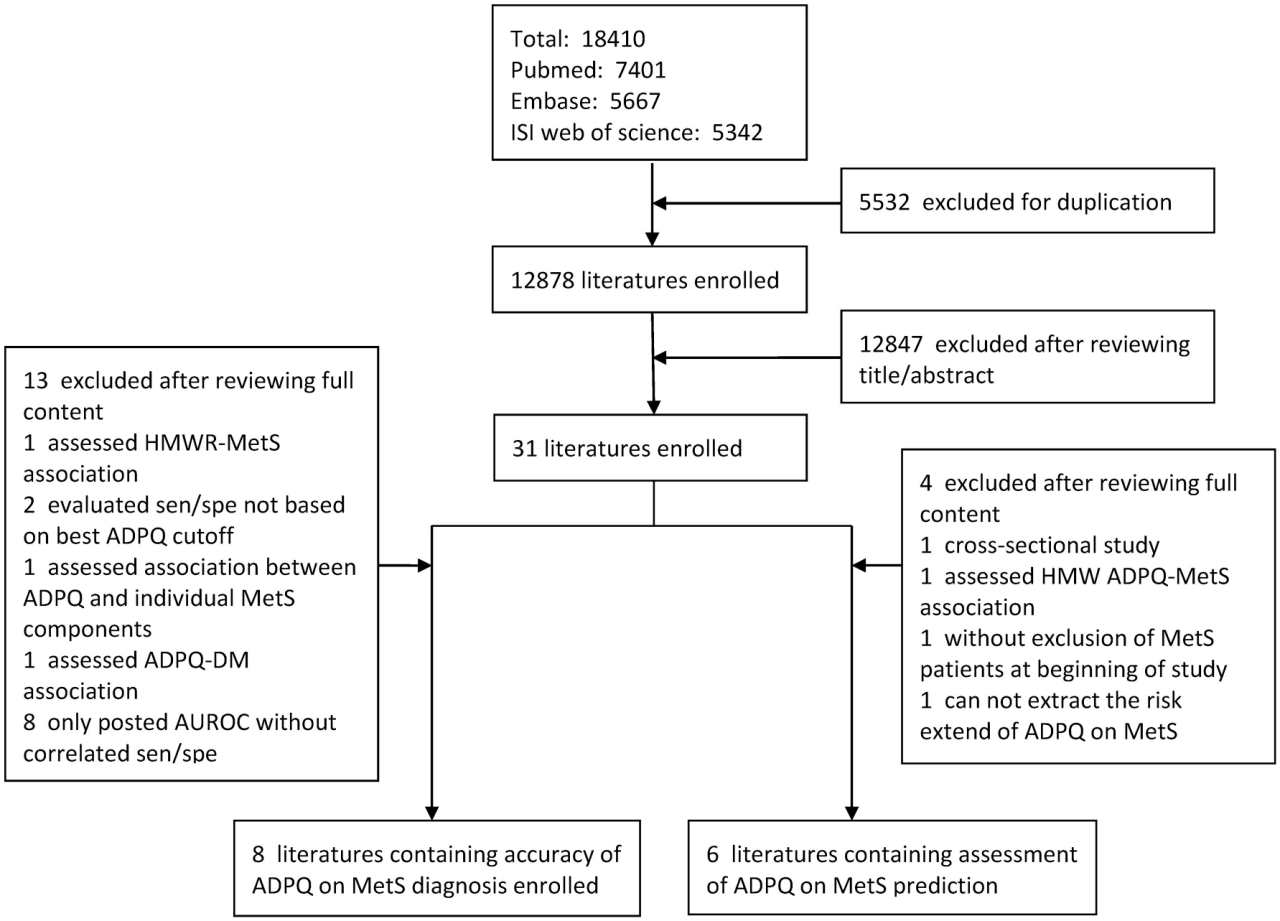
Flow diagram of selection for qualified studies in meta-analysis. AUROC, area under the receiver operating characteristic; DM, diabetes mellitus; HMW ADPQ, high-molecular weight adiponectin; MetS, metabolic syndrome; SEN, sensitivity; SPE, specificity.

The literature search procedure is presented in Figure 1. In total, 18410 original studies were obtained based on a predesigned search strategy (Table S1). Unpublished studies were not considered for inclusion. After removing 5,532 duplicates, 30 studies were selected by reviewing titles and abstracts. Thirteen studies were identified to fulfill the eligibility criteria and were included in the final analysis. Among enrolled literature, six studies (Ogawa et al., 2005; Gilardini et al., 2006; Mojiminiyi et al., 2007; Lee et al., 2008; Boyraz et al., 2013; Patel et al., 2015) focused on diagnostic accuracy; five studies (Seino et al., 2009; Juonala et al., 2011; Nakashima et al., 2011; Kim et al., 2013a; Lindberg et al., 2017) focused on longitude risk extent; and one study (Hata et al., 2015) focused both on topics referred above.

### Quality assessment

Assessment of methodological quality with regard to diagnostic accuracy of ADPQ upon MetS diagnosis was performed based on the Quality Assessment of Diagnostic Accuracy Studies-2 (QUADAS-2) tool. In general, enrolled studies have high quality for low risk in each item of bias assessment. One study (Patel et al., 2015) had a high risk of bias for the adoption of predesigned cut-off values. Another study (Mojiminiyi et al., 2007) exhibited a high risk of bias for the enrollment of diabetic patients (Table S2). The quality of each item for all studies based on the QUADAS-2 tool is summarized in Figure S2.

To assess MetS risk based on ADPQ variation, the modified Newcastle-Ottawa scale (NOS) scale was adopted for quality assessment (Supplementary NOS Checklist). All enrolled studies scored ≥6 and were considered to exhibit high quality regarding reliability and validity (Table S3). With respect to factors with low evidence quality identified in individual studies, two studies (Seino et al., 2009; Hata et al., 2015) did not adjust potential confounders in the risk assessment. Two studies (Kim et al., 2013a; Hata et al., 2015) had defects regarding outcome assessment, specifically, a short follow-up duration or lower response rate.

### Characteristics of enrolled studies

The features of enrolled studies regarding accuracy test and risk assessment are described in Tables 1, 2, respectively. One study (Hata et al., 2015) assessed both accuracy and prediction.

**Table 1:** Characteristics of enrolled studies for accuracy of circulating adiponectin level on metabolic syndrome diagnosis.

		**Population**			**Adiponectin**		**MetS**		**Accuracy**			
**Data source (Author, year, country)**	**Study design**	**Charac-teristics**	**Age**	**Gender (F/M)**	**Sample**	**Assay**	**Diagnosis**	**Prevalence (*N*, %)**	**Sensitivity**	**Speciality**	**Cut-off (ug/ml)**	**AUROC**
Ogawa et al., 2005, Japan	Cross-sectional	1. Obese adolescents, 2. Without DM, renal and here-ditary disease, 3. Without smoking and regular medication	11.0 ± 1.3	0/100	Serum	RIA	Japanese Criteria	39(39.0)	0.64	0.67	6.65	0.67 ± 0.06
Gilardini et al., 2006, Italy	Cross-sectional	1.Obese adolescents	14(9–18)	96/66	Serum	ELISA	WHO definition	M:20(30.3)	0.82(0.48–0.98)	0.57(0.39–0.74)	8.1	0.70 ± 0.05
								F:20(20.8)	0.77(0.46–0.95)	0.79(0.67–0.88)	8.3	0.87 ± 0.05
Mojiminiyi et al., Kuwait 2007	Cross-sectional	T2DM patients	MetS-M: 60(50–66) F:62(56–66) Non-MetS- M:65(59–70) F: 59 (54–65)	78/57	Plasma	ELISA	AHA/NHLBI criteria	M:36(63.2)	0.83	0.65	18	0.74 (0.64–0.84)
								F:46(59.0)	0.92	0.41		
Lee et al., USA, 2008	Cross-sectional	1. obese adolescents without medication affecting lipid and BP; 2. with less (< 10%) PCOS and DM cases	AA:12.7 ± 0.2	AA: 74/48	Serum	RIA	Modified NCEP-ATP-III criteria for children	AA:23(18.9)	0.83	0.82	AA: 10.8	0.89
			CA: 13.1 ± 0.2	CA: 71/58				CA: 24(18.6)	0.79	0.79	CA: 11.0	0.86
Boyraz et al., Turkey, 2013	Cross-sectional	1. Obese pubertal children, 2. without medication for DM or dyslipide-mia	M:12.5 ± 1.9 F:12.2 ± 2.3	85/63	Serum	ELISA	Modified WHO criteria for children	35(23.6)	0.74	0.81	2.85	0.82(0.74–0.90)
Hata et al., Japan, 2015	Pros-pective study	1. General population, 2. without MetS at baseline	40.4 ± 0.5	0/365	Serum	RIA	Interim criteria	45(12.3)	0.73	0.62	6.2	NA
Patel et al., India, 2015	Cross-sectional	1. General population, 2. Without ischemic, renal, liver diseases and cancer, 3. Without taking alcohol, tobacco and medication for chronic disease.	MetS: 50.7 Non-MetS: 49.3	41/46	Serum	ELISA	Interim criteria	44(50.6)	0.73	0.56	17.7	0.68(0.58–0.76)

*AA, African American; AUCROC, area under the receiver operating characteristic; CA, Caucasian; ELISA, enzyme-linked immunoabsorbent assay; F, female; M, male; MetS, metabolic syndrome; RIA, radioimmuno assay; T2DM, type 2 diabetes mellitus*.

**Table 2:** Characteristics of enrolled studies on risk assessment of blood adiponectin level on metabolic syndrome incidence.

**Data source (Author, country, year)**	**Population**			**Study**	**Adiponectin**			**MetS**		**Risk assessment**				
	**Base-line Features**	**Age (year)**	**Gender (F/M)**	**Follow up (year)**	**Category**	**Sample**	**Measurement**	**Definition**	**Incidence (N, %)**	**Indicator**	**Comparison (μg/ml)**	**Extent**	**Methods**	**Adjusted covariates**
Seino et al., Japan, 2009	1. General population 2. Without MetS, endocrine disease, renal or hepatic disease 3. Without medication for diabetes	MetS: 46.6 ± 9.2	0/416	6	Total	Serum	ELISA	Japanese criteria	27, 6.4	OR	T1 vs. T3 (≥7.44 vs. ≤ 4.65)	0.45 0.17–1.19)	Chi-square	NA
		Non-MetS: 44.1 ± 8.3			HMW					HR	≤ 2.65 vs. >2.65	1.56 (1.05–2.29)	Cox hazard model	Age and BMI
										OR	T1 vs.T3 (≥5.07 vs. ≤ 3.16)	0.24 (0.08–0.75)	Chi-square	NA
Nakashima et al., Japan, 2011	1. General population 2. without MetS and DM	Non-MetS (M): 61.2 ± 16.0	312/224	3.2	Total	Plasma	ELISA	AHA/NHLBI	M: 46,20.5	HR	M: SD (5.7) incre-ment vs. before	0.68 (0.51–0.92)	Cox hazards model	NA
		Non-Mets (F): 59.8 ± 13.2							F: 43, 13.8	HR	F: SD (6.6) incre-ment vs. before	0.63 (0.46–0.87)	Cox hazards model	NA
		Men MetS (M): 60.8 ± 14.8			HMW					HR	M: SD (4.3) incre-ment vs. before	0.69 (0.51–0.93)	Cox hazards model	Age, BMI, OGTT, and HOMA-IR
		MetS (F): 63.3 ± 10.4								HR	F: SD (5.5) incre-ment vs. before	0.70 (0.51–0.96)	Cox hazards model	Ditto
Juonala et al., Finland, 2011	1. General population 2. without MetS	Non-MetS: 31.5 ± 5.0	659/829	6	Total	Serum	RIA	NCEP-ATPIII	M: 102, 12.3	OR	1 unit increment vs. before	0.94 (0.90–0.99)	Logistic regression	Age, sex, BMI, LDL-C, CRP,
		MetS: 33.0 ± 4.7							F: 133, 20.2					leptin, insulin, smoking, family history of CVD, WC, HDL-C, TG, SBP and FBG
Kim et al., Korea, 2013b	1. General Population 2. without any MetS components 3. without CAD history	MetS (M): 56.0 ± 8.1	1,231/831	2.6	Total	Serum	RIA	Modified NCEP-ATP-III	M: 153, 18.4	OR	M: Q4 vs. Q1 (>11.22/ < 5.94)	0.25 (0.14-0.47)	Logistic regression	Age, BMI, LDL-C, smoking, exercise, CRP, HOMA-IR and TG.
		Non-MetS (M): 56.6 ± 8.2							F: 199, 16.2	OR	M: 5 unit incre-ment vs. before	0.82 (0.73–0.92)	Logistic regression	DITTO
		MetS (F): 55.4 ± 7.8								OR	F: Q4 vs. Q1 (>15.24/ < 8.91)	0.45 (0.28–0.74)	Logistic regression	DITTO
		Non-MetS (F): 52.5 ± 7.9								OR	F: 5 unit incre-ment vs. before	0.90 (0.83–0.96)	Logistic regression	DITTO
Hata et al., Japan, 2015	1. General population 2. Without MetS	40.4 ± 0.5	0/365	3.1	Total	Serum	RIA	IDF	45, 12.3	OR	Q4 vs Q1 (≥8.9 vs. ≤ 4.9)	0.14 (0.04–0.48)	Chi-square	NA
Lindberg et al., Denmark, 2017	1. General population 2.Without MetS, CVD, DM 3. Without medication on MetS	Q1:42 ± 8	406/747	9.4	Total	Plasma	Immuno-fluoro-metric assay	NCEP-ATP-III criteria	187,16.2	OR	Q1 vs. Q4 (≤ 6.6 vs. >12.5)	2.24 (1.11–4.52)	Logistic-regression	Age, gender, smoking, physical activity, SBP, DBP, FBG, BMI, TC, HDL, LDL, and eGFR.
		Q2:44 ± 8								OR	Per 1 unit decre-ment vs. before	1.56 (1.10–2.23)	Logistic-regression	DITTO
		Q3:45 ± 9												
		Q4:49 ± 8												

*BMI, body mass index; CRP, C-reactive protein; CVD,cardiovascular disease; DBP, diastolic blood pressure; eGFR, estimated glomerular filtration rate; ELISA, enzyme-linked immunoabsorbent assay; F, female; FBG, fasting blood glucose; HDL-C, high density lipoprotein cholesterol; HOMA-IR, homeostasis model assessment of insulin resistance; HR, hazard ratio; LDL-C, low density lipoprotein cholesterol; M, male; MetS, metabolic syndrome; NA, not available; OGTT, oral glucose tolerance test; OR, odds ratio; Q, quantile; RIA, radioimmuno assay; SBP, systolic blood pressure; SD, standard deviation; TC, total cholesterol; TG, triglyceride; WC, waist circumference*.

Regarding the validity of ADPQ measurements with respect to the diagnosis of MetS, greater than 1248 subjects (803 males and 445 females, with 26.6% MetS prevalence) were included in the final analysis. Adolescent (mean age range: 11–14 years) and adult participants with various backgrounds (diabetes mellitus [DM] or general subjects with ages ranging from 40 to 65 years) were involved in four and three studies, respectively. Obese participants were enrolled in most (three in four) adolescent studies.

The state of insulin resistance was reported or can be evaluated via calculation in a majority of the studies except one (Hata et al., 2015). Extremely severe insulin resistance was noted in adolescents from one US study (Homeostatic Model Assessment for Insulin Resistance [HOMA-IR] index: 8 and 7.6 for African-Americans and Caucasians, respectively) (Lee et al., 2008) compared with the remaining studies (ranged between 2.13 and 4.83). Studies were cross-sectional (*n* = 6) and prospectively (*n* = 1) designed. Total ADPQ levels were measured in serum (*n* = 6) or plasma (*n* = 1) and assessed by enzyme-linked immunoabsorbent assay (ELISA, *n* = 4) or radioimmunoassay (RIA, *n* = 3). In all adolescent studies, serum was sampled for ADPQ measurements.

For risk assessment of ADPQ and MetS incidence, six prospective studies including 6,020 general adults were included in the final analysis. Qualified participants included 3,412 men and 2,608 women aged between 31 and 63 years with similar average body mass index (BMI) of approximately 22.8–24.2 kg/m^2^. The HOMA-IR index could not be obtained in two studies (Hata et al., 2015), and the value was similar among the other three studies (ranged between 1.16 and 1.56). Four studies with 3,996 participants and corresponding 19,789.79 person-years in follow-up duration were extracted to assess linearity and dose-response risks. The MetS incidence differed across individual studies (6.4–20.5%) with varied in follow-up durations (2.6–9.4 years). The average MetS incidence rate was 32.5 cases per 1,000 person years in all studies (30.7 for men and 46.0 for women). All studies measured the total ADPQ value in serum (*n* = 4) or plasma (*n* = 2) samples using ELISA (*n* = 2) or RIA (*n* = 4) methods. Additionally, two studies (Seino et al., 2009; Nakashima et al., 2011) also tested high-molecular-weight (HMW) ADPQ, which is considered a more sensitive predictor in screening MetS progression (Hara et al., 2006). All studies reported the odds ratio (OR, *n* = 4), hazard ratio (HR, *n* = 1) or both (*n* = 1) as indicators to estimate the risk measures of MetS occurrence based on ADPQ variation. Risk trends followed with varied ADPQ categories in each individual study are presented in Figure S3.

The MetS definitions for all enrolled studies (*n* = 12) are summarized in Table S4. For adults, most studies (6 in 8) adopted joint interim criteria (Grundy et al., 2005; Alberti et al., 2009). The remaining two studies used criteria defined by the National Cholesterol Education Program Adult Treatment Panel III (NCEP-ATP-III) (Expert Panel on Detection, 2001) and a Japanese committee (Matsuzawa, 2005). For adolescents, definitions from the World Health Organization (Organization, 1999) and NCEP criteria for children (Weiss et al., 2004) were applied in two studies and one study, respectively. One Japanese study adopted self-defined criteria.

The ADPQ value categorized by MetS status was presented in studies related to diagnostic accuracy (*n* = 5) and risk assessment (*n* = 4). The average ADPQ levels in each MetS group were less than corresponding values from the non-MetS group (Figure S4). In total, ADPQ values was reduced significantly in MetS compared to non-MetS subjects, no matter in groups regarding diagnostic accuracy (8.94 ± 5.84 vs. 10.44 ± 6.33 ug/ml) or risk prediction (9.18 ± 4.96 vs. 9.88 ± 5.00 ug/ml, both *P* < 0.01).

### Accuracy of ADPQ in MetS diagnosis

**Figure 2:**
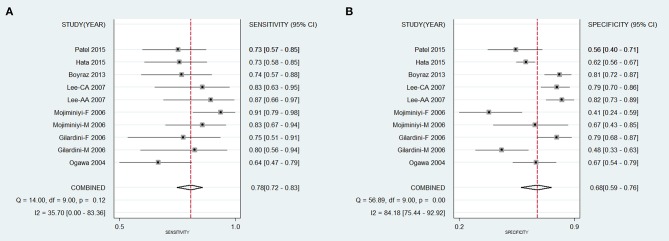
Pooled sensitivity and specificity for accuracy of circulating adiponectin upon diagnosis of metabolic syndrome. **(A)** Pooled sensitivity of ADPQ upon MetS diagnosis; **(B)** Pooled specificity of ADPQ upon MetS diagnosis. AA, African American; ADPQ, adiponectin; CA, Caucasian; F, female; M, male; MetS, metabolic syndrome.

**Figure 3:**
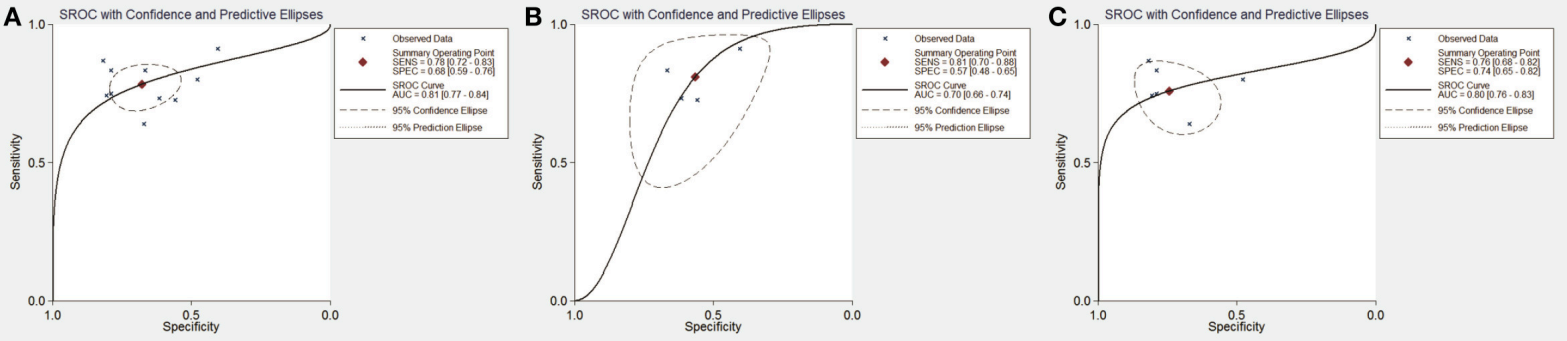
Summary receiver operating characteristic curve for test accuracy of adiponectin upon metabolic syndrome diagnosis. **(A)** SROC curve for accuracy of ADPQ upon MetS diagnosis in all cohorts; **(B)** SROC curve for accuracy of ADPQ upon MetS diagnosis in adult cohorts; **(C)** SROC curve for accuracy of ADPQ upon MetS diagnosis in adolescent cohorts. ADPQ, adiponectin; SROC, summary receiver operating characteristic; MetS, metabolic syndrome.

The diagnostic accuracy of the ADPQ assay in MetS diagnosis was based on ten data clusters extracted from all seven extractable studies. As shown in Figure 2, the pooled sensitivity and specificity were 0.78 (95% CI: 0.72–0.83) and 0.68 (95% CI: 0.59–0.76), respectively. Correspondingly, the pooled PLR, NLR, and DOR were 2.44 (95% CI: 1.87–3.18), 0.32 (95% CI: 0.25–0.42), and 7.62 (95% CI: 4.78–12.2), respectively. NPV and PPV were 0.90 and 0.47, respectively, based on average MetS prevalence (26.6%) in enrolled studies (Table S5). The area under the hierarchical summarized ROC curve was 0.81 (95% CI: 0.77–0.84, Figure 3A).

Regarding heterogeneity observed across enrolled studies, the *I*^2^ values for pooled SPE (84.2%), PLR (71.6%), and DOR (45.5%) were increased compared with SEN (35.7%) and NLR (39.3%). Different cut-off values across enrolled studies did not influence the test accuracy for an insignificant threshold effect of ADPQ on MetS diagnosis (*P* = 0.841). Subgroup analysis was performed to investigate the impact of potential confounders on overall effects (Table 3). For SEN, the heterogeneity was significantly decreased in studies with adolescents, lower ADPQ cutoff value, and low MetS prevalence (all *I*^2^ < 25%). SEN was significantly increased in a cohort of DM patients (Lee et al., 2008) using plasma samples for ADPQ measurement (0.88 vs. 0.75, *P* = 0.04) with low heterogeneity (*I*^2^ < 25%). With respect to SPE, decreased heterogeneity was only observed in the male subgroup (*I*^2^ = 0%). Increased SPE was observed in the adolescent cohort (0.76 vs. 0.60, *P* = 0.01). As a joint effect of SEN and SPE, the summary DOR (sDOR) was associated with sample selection, DM status, and HOMA-IR index (*P* < 0.05). A multi-covariate meta-regression model found that ADPQ measurements in cohorts with higher insulin resistance (HOMA-IR > 5) exhibited better performance in MetS diagnosis (ratio of DOR [rDOR] = 3.89, 95% CI: 1.13–13.39, *P* < 0.05).

**Table 3:** Subgroup analysis for accuracy of adiponectin test on diagnosis of metabolic syndrome.

		**Sensitivity**			**Specificity**			**Joint effect**
	***N***	**Mean (95%CI)**	***I*^2^(%)**	***P***	**Mean (95%CI)**	***I*^2^(%)**	***P***	***P***
**AGE**								
< 18	6	0.76(0.68–0.82)	9.1		0.76(0.72–0.80)	66.5		
>18	4	0.80(0.73–0.86)	58.4	0.52	0.60(0.55–0.64)	49.0	0.01	0.08
**GENDER**								
Male	4	0.74(0.66–0.81)	25.8		0.62(0.57–0.67)	0		
Female	2	0.86(0.76–0.94)	65.5		0.68(0.58–0.76)	93.2		
Mixed	4	0.78(0.70–0.85)	0	0.78	0.78(0.73–0.82)	74.5	0.20	0.11
**DM COHORT**								
Yes	2	0.88(0.79–0.94)	15.9		0.51(0.37–0.65)	71.4		
No	8	0.75(0.69–0.80)	0	0.04	0.70(0.67–0.73)	82.2	0.17	0.02
**BASELINE ADPQ VALUE (ug/ml)**								
< 10	5	0.74(0.66–0.81)	12.6		0.68(0.65–0.72)	85.2		
>10	5	0.82(0.75–0.87)	35.2	0.24	0.70(0.64–0.75)	82.8	0.79	0.38
**BASELINE HOMA-IR**								
< 5	8	0.77(0.70–0.83)			0.63(0.53–0.73)			
>5	2	0.85(0.72–0.94)	0	0.33	0.80(0.74–0.86)	0	0.07	0.03
**MEAN BMI**								
< 30	6	0.76(0.70–0.81)	26.8		0.68(0.64–0.71)	79.7		
>30	4	0.82(0.74–0.88)	41.7	0.38	0.71(0.65–0.77)	88.0	0.66	0.54
**SAMPLE SIZE**								
< 100	5	0.81(0.75–0.87)	35		0.63(0.56–0.69)	77.1		
>100	5	0.82(0.76–0.87)	21.2	0.30	0.65(0.59–0.71)	79.3	0.08	0.09
**ADPQ CUT-OFF (ug/ml)**								
< 10	5	0.72(0.65–0.79)	0		0.67(0.64–0.71)	81.7		
>10	5	0.83(0.77–0.89)	31.1	0.07	0.72(0.66–0.77)	85.2	0.92	0.10
**MetS PREVALANCE (%)**								
< 35	6	0.78(0.71–0.84)	0		0.71(0.68–0.74)	85.7		
>35	4	0.78(0.71–0.84)	72.8	0.98	0.59(0.51–0.66)	55.9	0.13	0.20
**SPECIMEN**								
Serum	8	0.75(0.69–0.80)	0		0.70(0.67–0.73)	82.2		
Plasma	2	0.88(0.79–0.94)	15.9	0.04	0.51(0.37–0.65)	71.4	0.17	0.02
**METHODS**								
ELISA	6	0.80(0.74–0.85)	28.7		0.69(0.64–0.74)	82.7%		
RIA	4	0.75(0.66–0.82)	43.7	0.45	0.69(0.65–0.73)	86.2%	0.37	0.42

*ADPQ, adiponectin; BMI, body mass index; DM, diabetes mellitus; ELISA, enzyme-linked immunoabsorbent assay; HOMA-IR, homeostasis model assessment of insulin resistance; MetS, metabolic syndrome; RIA, radioimmuno assay*.

In adolescents, ADPQ had reproducible diagnostic performance on MetS in obese subjects after excluding a cohort with an increased HOMA-IR index (Lee et al., 2008) (sDOR = 6.99, 95%CI: 3.76–12.98, *I*^2^ = 23.6%). In adults, the accuracy of ADPQ in MetS diagnosis was also significant (sDOR = 4.91, 95%CI: 3.08–7.84), but unstable effects were noted for mixtures of DM and normal subjects. Inconsistencies in sensitivity and specificity were reduced after excluding the cohort with adult DM patients (both *I*^2^ = 0%). Compared with adolescents, significantly reduced AUC of ADPQ for MetS diagnosis was observed in adult cohorts (0.70 vs. 0.83, *P* < 0.05, Figures 3B,C).

### Dose-response risk assessment of ADPQ in predicting MetS incidence

**Figure 4:**
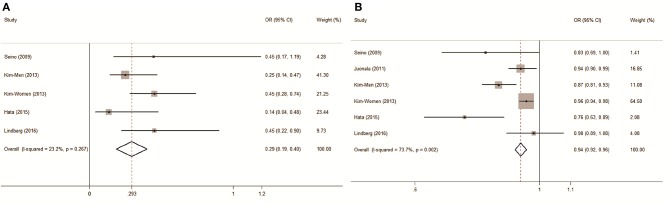
Forest plot on association between circulating adiponectin and metabolic syndrome risk assessed in prospective studies. **(A)** Pooled OR of MetS compared between groups with highest and lowest ADPQ levels; **(B)** Pooled OR of MetS followed per 1 ug/ml ADPQ increase. ADPQ, adiponectin; MetS, metabolic syndrome; OR, odds ratio.

As a biomarker for MetS prediction in a longitudinal study, the pooled OR of incident MetS compared between subjects with the highest and lowest ADPQ values was 0.29 (95% CI: 0.19–0.40) with low inter-subgroup heterogeneity (*I*^2^ = 23.2%, *P* = 0.267) based on five data points incorporated from four studies (Figure 4A).

**Figure 5:**
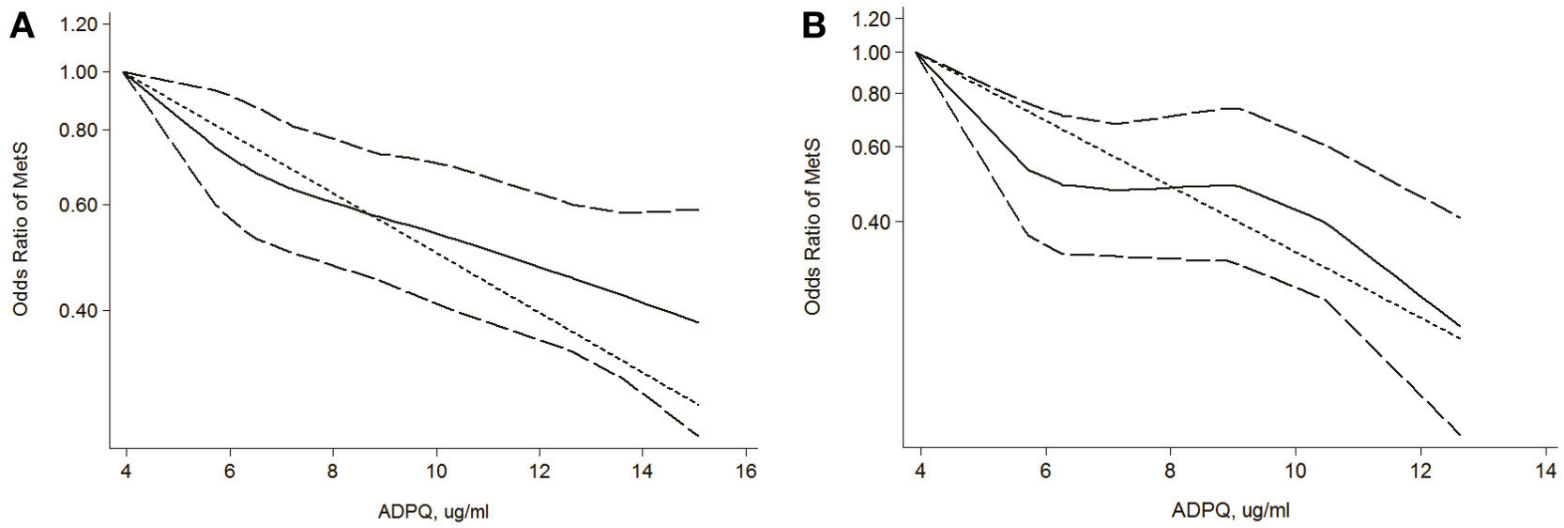
Dose-response relationship between circulating adiponectin levels and metabolic syndrome risk. **(A)** Evaluation of association between circulating ADPQ and MetS risk in all cohorts based on restricted cubic splines and generalized least squares dose-response models; **(B)** Evaluation of association between circulating ADPQ and MetS risk in male cohorts based on restricted cubic splines and generalized least squares dose-response models. The solid and long-dashed curves represent instant estimates of the OR and its 95% CI for MetS risk based on the subgroup with the lowest average ADPQ level based on the restricted cubic splines model. The short-dashed line represents the estimates of the OR for MetS risk based on the subgroup with lowest average ADPQ level using the generalized least squares model. ADPQ, adiponectin; CI, confidence interval; MetS, metabolic syndrome; OR, odds ratio.

An approximately 2.0 cases per 1,000 person-years reduction in MetS risk was associated with a 1 ug/ml increase ADPQ with moderate inconsistency across individual studies (pooled OR: 0.94, 95% CI: 0.92–0.96; *I*^2^ = 73.7%, *P* = 0.002, Figure 4B). However, we did not detect non-linearity in the dose-response relationship between blood ADPQ value and MetS incidence based on restricted cubic spline models (*P* = 0.42, Figure 5A). The ADPQ-MetS association tended to fit linear trend with significant inter-subgroup heterogeneity (*P* = 0.009) based on a random-effects meta-regression model corrected by generalized least squares (GLS) estimates (*P* < 0.01). For data exclusively including men, there was no evidence of a non-linear relationship between circulating ADPQ and MetS (*P* = 0.12, Figure 5B), but obvious inter-study heterogeneity was not noted (*P* = 0.19). Similarly, the linear trend for the ADPQ-MetS association was also significant in men based on GLS methods in fixed effect meta-regression models (*P* < 0.01). An approximately 4.6 cases per 1,000 person-year reduction in MetS incidence was associated with a 1 ug/ml increase in ADPQ in male populations (pooled OR = 0.85, 95% CI: 0.80–0.90).

Potential confounders of the dose-response MetS risk were investigated by stratification analysis (Table 4). Age, ethnicity, follow-up duration, measurement (sample/method), MetS incidence and definition did not cause significant inconsistencies in final results (*P* > 0.05). The reduced OR in male cohorts compared with females partially contributed to the heterogeneity (0.85 vs. 0.96, *P* < 0.01). Intriguingly, a prominent decrease in the dose-response MetS risk was observed in cohorts with lower baseline ADPQ levels that monotonically comprised by men. Further Mendelian randomization analysis revealed the internal differentiation for gender originated from increased susceptibility of IR (OR = 1.07, 95%CI: 1.04–1.09) in men independent of its specific reduction in ADPQ values (SMD = −1.05, 95% CI: −1.12/−0.97 ug/ml). The pooled OR was significantly reduced in two Japanese cohorts (Seino et al., 2009; Hata et al., 2015) with lower MetS incidence (*P* = 0.002). However, the lower MetS risk might result from the lack of BMI adjustment in these studies.

**Table 4:** Subgroup analysis for risk assessment of adiponectin on metabolic syndrome incidence.

	***N***	**OR (95%CI)**	**I^2^**	**P${}_{1}^{\text{a}}$**	**P${}_{2}^{\text{b}}$**
**AGE (YEARS)**					
< 45	3	0.91(0.87–0.95)	75.1%	0.018	
≥45	3	0.95(0.93–0.97)	77.8%	0.011	0.158
**GENDER**					
Male	3	0.85(0.80–0.90)	17.6%	0.297	
Female	1	0.96(0.94–0.98)	NA	NA	
Mix	2	0.95(0.91–0.99)	0%	0.419	< 0.01
**ETHNICITY**					
Caucasian	2	0.95(0.91–0.99)	0%	0.419	
East Asian	4	0.94(0.92–0.96)	83.5%	< 0.001	0.660
**FOLLOW-UP (YEARS)**					
< 4	3	0.94(0.92–0.96)	87.7%	< 0.001	
>4	3	0.94(0.90–0.98)	28.6%	0.246	0.979
**SAMPLE**					
Serum	5	0.94(0.92–0.96)	78%	0.001	
Plasma	1	0.92(0.89–1.07)	NA	NA	0.360
**METHOD**					
RIA	4	0.94(0.92–0.96)	81.5%	0.001	
ELISA	2	0.94(0.86–1.02)	63.4%	0.094	0.954
**MeTS INCIDENCE**					
< 15%	2	0.79(0.69–0.89)	0%	0.508	
>15%	4	0.95(0.93–0.96)	67.0%	0.028	0.002
**MeTS DEFINITION**					
NCEP-ATP-III	2	0.95(0.91–0.99)	0%	0.419	
Modified NCEP-ATP-III	3	0.94(0.92–0.96)	87.1%	< 0.001	
Other definitions	1	0.83(0.67–0.98)	NA	NA	0.341
**BMI ADJUSTMENT**					
Yes	4	0.95(0.93–0.96)	67.0%	0.028	
No	2	0.79(0.69–0.89)	0	0.508	0.002
**BASELINE ADPQ VALUE (mg/L)**					
< 9	3	0.85(0.80–0.90)	17.6	0.297	
≥9	3	0.96(0.94–0.98)	0	0.663	< 0.01

a *P_1_ value represents heterogeneity in subgroups*.

b *P_2_ value represents heterogeneity across subgroups*.

*ADPQ, adiponectin; BMI, body mass index; ELISA, enzyme-linked immunoabsorbent assay; MetS, metabolic syndrome; NA, not available; NCEP-ATP-III, National Cholesterol Education Program Adult Treatment Panel III; OR, odds ratio; RIA, radioimmuno assay*.

Results from sensitivity analysis also identified that the pooled results exhibited an approximately 3% reduction compared with the primary overall results after omitting the only female study by Kim et al. (2013a) (0.91 vs. 0.94, *P* = 0.011, Figure S5). In addition, no individual study significantly influenced the pooled dose-response OR with ADPQ variation (pooled OR range: 0.94–0.95, all *P* > 0.05).

### Publication bias

For diagnostic accuracy, the funnel plot exhibited a symmetrical shape (Figure S6A). The results from asymmetry tests also supported insignificant publication bias across individual studies (*P* = 0.379). For risk assessment, convincing evidence indicating significant publication bias across different studies was not revealed based on Egger and Begg's test (*P* = 0.103 and 0.260, respectively) despite the slight asymmetry observed in the funnel plot (Figure S6B).

## Discussion

To be best of our knowledge, this is the first evidence-based study to comprehensively analyze the accuracy and predictive validity of circulating ADPQ in MetS detection. Compared with normal subjects, all included studies reported lower average ADPQ values in the corresponding MetS group (Figure S4), indicating their undoubted close relationship. As a reliable estimate for adipocyte function, ADPQ was also recommended to be combined with HbA1c for early diagnosis of DM in moderate accuracy (AUROC = 0.85) in previous study (Kälsch et al., 2015). However, the accuracy and predictive validity of ADPQ as a single indicator for MetS remained debated across populations with different backgrounds. Based on published literature, the ADPQ test is able to distinguish MetS patients from normal subjects with moderate diagnostic accuracy (pooled AUROC = 0.81, 95% CI: 0.77–0.84). Pubertal development and metabolic factors (including insulin resistance and obesity) might contribute to the heterogeneity of its accuracy.

It was noteworthy that stratified analysis revealed an increased diagnostic value for ADPQ measurement in specific populations, such as DM patients and obese adolescents. As a key covariate, HOMA-IR should be considered for adjustment in this evaluation. With respect to the predictive validity, results from prospective studies with 6,020 participants revealed a negative association between adiponectin levels and MetS incidence in a linear dose-response fashion. Approximately 6 and 15% increases in MetS incidence were associated with a 1 ug/ml decrease in ADPQ in the entire cohort and males, respectively, which is equivalent to 2 and 4.6 fewer cases per 1,000 person years, respectively. Despite the relative lower ADPQ level, an increased risk for hypoadiponectinemia with MetS incidence in men could explain their intrinsic higher susceptibility (approximately 7%) for MetS occurrence.

Concerning the clear evidence of effects on alleviating insulin resistance (Yamauchi et al., 2001; Fu et al., 2005), ADPQ serves as a potentially valid biomarker to detect MetS in population studies. One study (Mojiminiyi et al., 2007) reported significant increases in the pooled SEN and DOR (*P* < 0.05), possibly due to its distinct sample selection (plasma) and diabetic status of participants upon subgroup analysis. However, heterogeneity tends to originate from DM, but not specimen for high reproducibility and stability of ADPQ as a secretory protein in both serum and plasma (Pischon et al., 2003; Yu et al., 2011). ADPQ has a close relationship with insulin resistance even after adjusting for central adiposity (Weyer et al., 2001). In our study, confounding effects of the diabetic status of participants on test accuracy might have originated from an extremely increased prevalence of insulin resistance (up to 84%) (Bonora et al., 1998) in patients with type 2 diabetes, which should be noted and adjusted in future diagnostic tests. A previous study reported an increased risk of coronary heart disease and related mortality based on an interaction with diabetes (Isomaa et al., 2001). It is worthy for risk stratification to distinguish MetS in patients with a diabetic background, and ADPQ measurements might be a suitable candidate for superior diagnostic value in this specific high-risk population. Correspondingly, approximately four-fold increased accuracy (rDOR = 3.89, 95%CI: 1.1–13.4, *P* < 0.05) in cohorts with increased insulin resistance (HOMA-IR > 5) also supported this hypothesis regarding insulin resistance as a potential confounder. The influence of insulin resistance on diagnostic value should be noted and adjusted in further clinical practice.

ADPQ is closely correlated with MetS in adolescents based on a common influence via the interaction between puberty and adiposity (Weiss et al., 2004; Punthakee et al., 2006). This relationship was also significant in non-obese subjects (Weyer et al., 2001; Abbasi et al., 2004). Consistently, we observed hierarchical variation in the diagnostic performance of ADPQ in MetS diagnosis in adults. ADPQ tests for MetS diagnosis appear to be more accurate in adolescents compared with adults based on increased false negative rates at the expense of a slight reduction in true positive rates (Table 3). The efficiency of ADPQ for MetS diagnosis was also noted in a cohort with a mixture of normal weight subjects (Lee et al., 2008), indicating its potential diagnostic value in distinguishing MetS in subjects across the entire BMI profile. In our study, we observed satisfactory and reproducible diagnostic values for ADPQ in MetS diagnosis in adolescent cohorts as evidenced by an increased AUC (0.80, 95% CI: 0.76–0.83). However, increased sDOR in the cohort with an increased HOMA-IR index (9.98 vs. 6.99, *P* < 0.05) indicates that key factors, such as insulin resistance, should be adjusted. For adults, the efficiency of the ADPQ test upon MetS diagnosis seems inferior for lower AUROC values (0.70, 95% CI: 0.66–0.74). The lower diagnostic accuracy might be attributed to the enrollment of normal subjects with a low risk of insulin resistance. Subgroup analysis cannot be performed due to high inconsistency and the lack of a description of insulin resistance in current adult cohorts. More studies in adults with complete baseline features (especially for covariates referred to insulin resistance) are required for better risk stratification using ADPQ in MetS diagnosis.

Prior studies found that modulation of ADPQ level and the susceptibility of insulin resistance might share a common genetic basis located in the human 3q27 locus (Vasseur et al., 2003; Yang and Chuang, 2006). Accordingly, key variations of the APM1 gene that encodes ADPQ protein were associated with MetS prevalence (Gao et al., 2013; Yuan et al., 2016). However, the causative role of ADPQ levels as an overall phenotype based on a gene-environmental interaction upon MetS occurrence has not been systematically reviewed to date. After pooling the published data, we observed a consistent and linear increase in MetS occurrence in subjects with hypoadiponectinemia. Significant heterogeneity was observed regarding the strength of this association (*I*^2^ = 73.7%, Figure 5). Stratification analysis revealed that gender might confound this causality (Figure 5, Figure S5, Table 4). Men seem more sensitive to insulin resistance, with approximately 2.3-fold increased MetS morbidity compared with entire cohorts in the same reduction in ADPQ levels.

Androgens modulate the ADPQ level by inhibiting its secretion from adipocytes to the circulation, and testosterone-related hypoadiponectinemia might contribute to specific severe insulin resistance in men (Nishizawa et al., 2002; Xu et al., 2005; Kadowaki et al., 2006). However, uncertainty still exists regarding whether the causal effects of this gender-specific correlation are based on hypoadiponectinemia in men or sexual dimorphism *per se*. Based on Mendelian randomization analysis, we observed inherently increased susceptibility of MetS (approximately 7%) in men independent of its lower baseline ADPQ level compared with women (SMD: −1.05 ug/ml). Therefore, significant lower OR of MetS incidence associated with ADPQ increased in cohorts with lower ADPQ levels at baseline (< 10 ug/ml, Table 4) might be better explained as concomitant hypoadiponectinemia with sexual dimorphism. Gender differentiation as a determinant of ADPQ-MetS causality should be taken into account in future longitudinal studies. Considering its potential therapeutic value (Montecucco and Mach, 2009), male cohorts might experience more benefits by improving ADPQ function and secretion and thus preventing MetS.

Centered on insulin resistance, a series of evidence-based studies revealed appreciable variation in ADPQ levels in patients with metabolic disorders. The pooled risks for diabetes and hypertension were approximately 0.72 and 0.94, respectively, with a 1 log transformed/normal unit increase in ADPQ (Li et al., 2009; Kim et al., 2013b). Even in patients with nonalcoholic steatohepatitis (NASH) as a hepatic manifestation of insulin resistance, the ADPQ value was significantly decreased (by approximately 2.6 mg/L) compared with normal subjects (Polyzos et al., 2011). The lower circulating ADPQ level was observed followed with increased NAFLD activity score (NAS) and histological severity (Wree et al., 2014). However, evidence is lacking for pooled effects of hypoadiponectinemia on MetS as a systemic presentation of insulin resistance (Eckel et al., 2005), key mediator and precursor of severe clinical complications (Wilson et al., 2005). Without any exception, lower ADPQ values in MetS patients were observed compared with non-MetS counterparts in each individual study (Figure S4), indicating the undoubted causality between ADPQ and MetS occurrence. Furthermore, we identified the superiority of the ADPQ test on MetS screening in high-risk populations with insulin resistance or adiposity. In addition, the predictive value was significantly affected by male-specific susceptibility to insulin resistance. These findings might be helpful to extend the utility of this biomarker for MetS prevention in further clinical practice.

Currently, ADPQ is considered as crucial adipocytokine in MetS development (Okamoto et al., 2006). Close hypoadiponectinemia–MetS association indicates impaired adipocyte function might significantly influence systemic insulin resistance (Stern et al., 2007; Klöting and Blüher, 2014). Revese effect of ADPQ on insulin resistance in animal and epidemiological studies (Yamauchi et al., 2001; Kim et al., 2015). was also indicative of its clinical implication and potential therapeutic value on MetS. Hence, ADPQ targeted pharmacologic intervention (e.g., recombinant adiponectin, statins, PPARγ agonist, endocannabinoid receptor antagonist et al.) and lifestyle modifications might (like exercise) have benefits on improvement of IR even CVD protection (Shetty et al., 2009; Wang et al., 2015). Consistent with previous studies, our results confirmed the closely negative dose-response relationship between ADPQ and MetS development. ADPQ might be a valuable target for MetS treatment, and extra benefits might be observed in high-risk subgroups like males or DM patients. Further clinical intervention studies are worthy to be performed to validate this viewpoint.

Our study identified ADPQ as a biomarker on detection of MetS and provided evidence about its causative role on the development of MetS. The contribution of ADPQ on the progression and occurrence of MetS can be studied through the use of systems biology tools (Bosley et al., 2017; Mardinoglu et al., 2018) where biological networks have been used in the analysis and integration of different omics data (Lee et al., 2017; Mardinoglu et al., 2017a; Uhlen et al., 2017). Recently, the mechanistic explanation in increased plasma mannose levels have been revealed through the use of biological networks (Lee et al., 2016) and it has been validated in large prospective cohorts (Mardinoglu et al., 2017b). Similar approaches can be also employed for revealing the mechanistic role of ADPQ on MetS development.

Certain limitations in our study should be noted. First, considering the gender differences, sex-specific diagnostic and predictive validity of ADPQ for MetS should be discussed separately. However, the dose-response OR cannot be pooled given the limited number of prospective studies in women (only one from a Korean population, Kim et al., 2013a). Second, potential bias might result from uncontrolled methodological quality in some studies. Third, ADPQ-related MetS risk might be overestimated for unadjusted OR by BMI and HOMA-IR. Heterogeneity caused by insulin resistance cannot be evaluated in the subgroup analysis given that the HOMA index was not provided in some original data (Hata et al., 2015; Lindberg et al., 2017). Fourth, although considered the active multimer of adiponectin with more relevance to protect against insulin resistance (Oh et al., 2007), the impact of high-molecular-weight ADPQ on MetS diagnosis and prediction cannot be pooled and compared with total ADPQ in less eligible publications (Seino et al., 2009; Nakashima et al., 2011). Fifth, differences in MetS definitions in cohorts with specific ethnicities and ages are noted across individual studies (Table S4). These differences might also cause inconsistency. However, insignificant heterogeneity was observed in subgroup analyses (Tables 3, 4).

In conclusion, our study revealed that circulating ADPQ might serve as an available diagnostic biomarker to identify MetS subjects, especially in high-risk populations with insulin resistance. Hypoadiponectinemia predicts increases in MetS incidence in a linear dose-response fashion, and gender should be taken into consideration in this evaluation. ADPQ might be an available target on MetS therapy, but the effects might be differentiated by patient feature.

## Author contributions

ZL and AM conceived and designed the study. ZL and SQ extracted information. SL analyzed the data. ZL wrote the manuscript. LZ, SZ, and AM reviewed the manuscript. All authors approved the final manuscript for submission.

### Conflict of interest statement

The authors declare that the research was conducted in the absence of any commercial or financial relationships that could be construed as a potential conflict of interest.
