# Supplementary material for: Meta-Analysis of Adiponectin as a Biomarker for the Detection of Metabolic Syndrome
Source: Front Physiol. 2018 Sep 19;9:1238. doi: 10.3389/fphys.2018.01238 (PMC6176651; doi:10.3389/fphys.2018.01238)
Supplement: Supplementary file 1 [file Data_Sheet_1.docx]

**Supplementary figures**


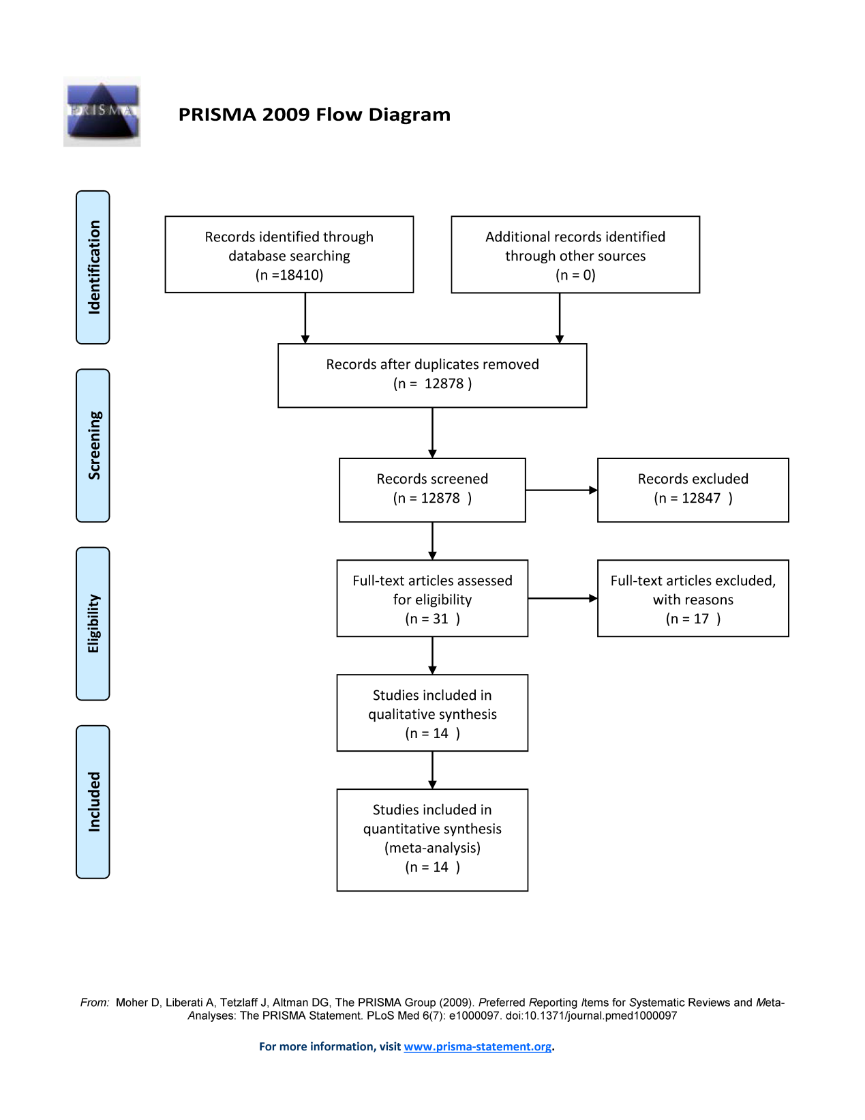


Figure S1 PRISMA Flow Diagram


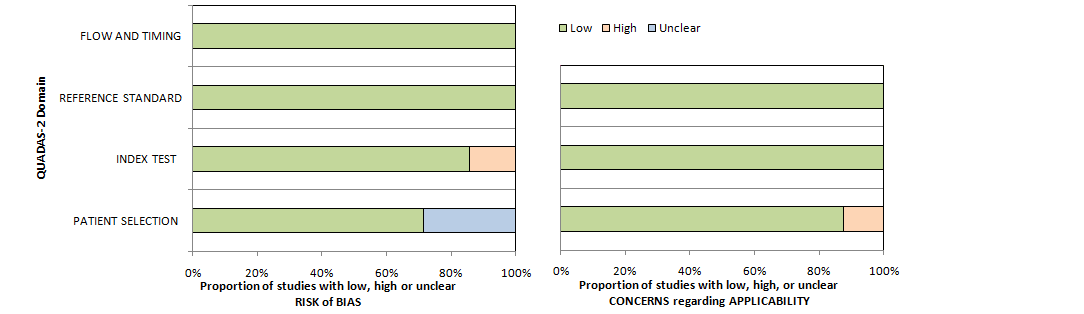


Figure S2 Quality assessment of diagnostic accuracy study based on QUADAS-2 scale

Abbreviations: QUADAS-2, Quality Assessment of Diagnostic Accuracy Studies-2.


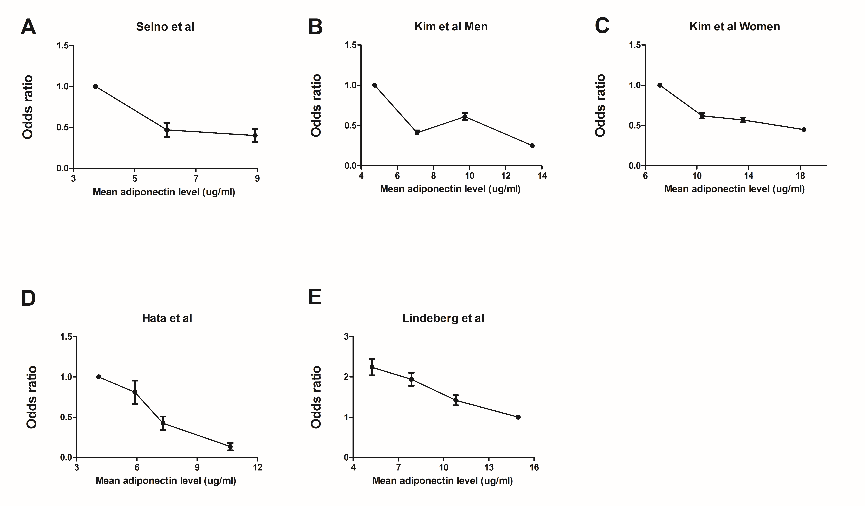


Figure S3 Trend for MetS risk followed with ADPQ variation in each individual studies

Abbreviations: ADPQ, adiponectin; MetS, metabolic syndrome.


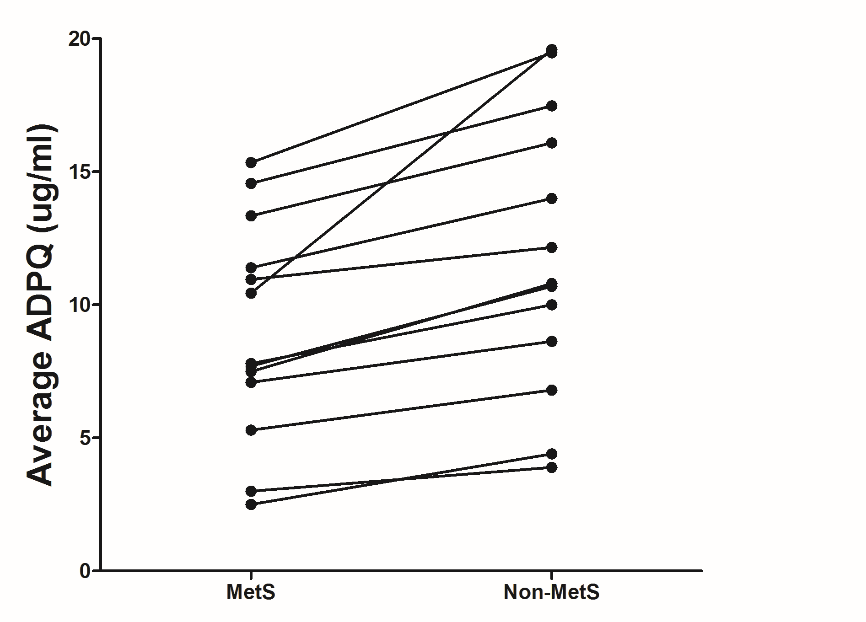


Figure S4 Comparison on average adiponectin level between MetS and non-MetS subgroups from each individual studies

Abbreviations: MetS, metabolic syndrome.


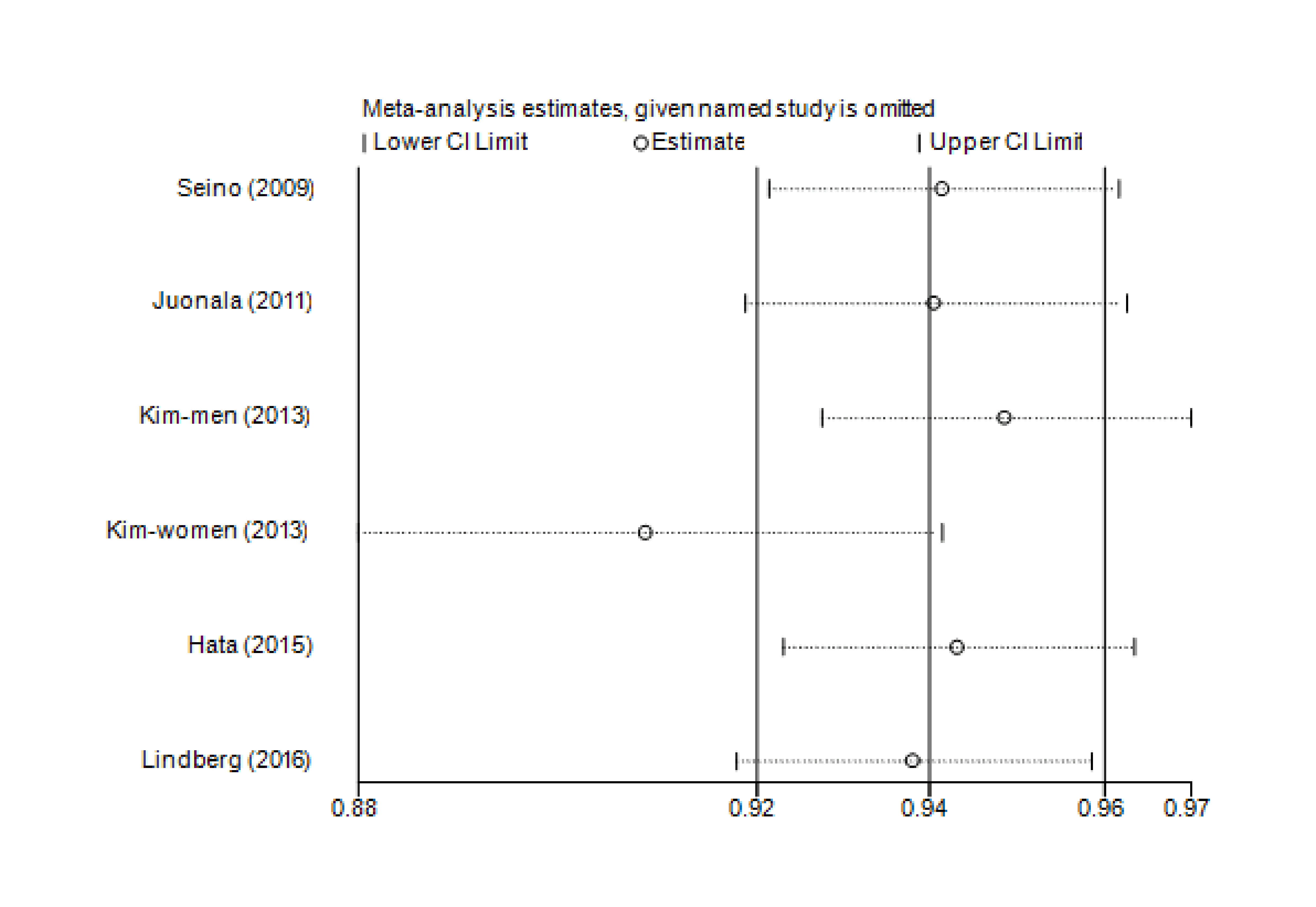


Figure S5 Sensitivity analysis on dose-responsed relationship between adiponectin level and metabolic syndrome incidence


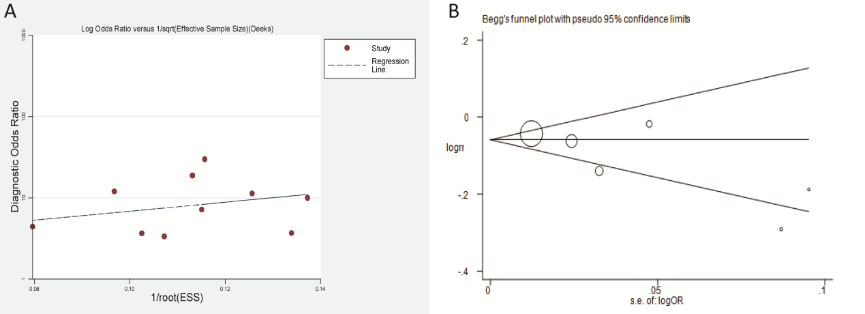


Figure S6 Publication bias on association between adiponctin and metabolic syndrome

A. Deek’s funnel plot asymmetry test on publication bias for accuracy of adiponectin on metabolic syndrome diagnosis;

B. Funnel plot for publication bias on odds ratios of hypoadiponectinemia with incident metabolic syndrome.

**Supplementary tables**

Table S1 Literature search strategy across different databases

| **ISI-web of science** |
| --- |
| *Indexes=SCI-EXPANDED, SSCI, A&HCI, CPCI-S, CPCI-SSH, ESCI, CCR-EXPANDED, IC Timespan=1990-2017* |
| #1 (TS=(adiponectin)) *AND* DOCUMENT TYPES: (Article) 15734 |
| #2 (TS=(ADIPOQ)) *AND* DOCUMENT TYPES: (Article) 581 |
| #3 (TS=(ADPQ)) *AND* DOCUMENT TYPES: (Article) 2 |
| \| #4 \| (TS=(ACDC)) *AND* DOCUMENT TYPES: (Article) 117 \| \| --- \| --- \|   #5 (TS=(GBP-28)) *AND* DOCUMENT TYPES: (Article) 1 |
| \| #6 \| \| (TS=(apM1)) *AND* DOCUMENT TYPES: (Article) 238 \| \| --- \| --- \| --- \| \| #7 \| (TS=(Acrp30)) *AND* DOCUMENT TYPES: (Article) 306 \| \|   #8 (TS=(metabolic syndrome)) *AND* DOCUMENT TYPES: (Article) 64623 |
| #9 (TS=(MetS)) *AND* DOCUMENT TYPES: (Article) 6116 |
| #10 (TS=(MS)) *AND* DOCUMENT TYPES: (Article) 361909 |
| #11 (TS=(syndrome X)) *AND* DOCUMENT TYPES: (Article) 31562 |
| #12 (TS=(insulin resistance syndrome)) *AND* DOCUMENT TYPES: (Article) 31311 |
| #13 (#1 OR #2 OR #3 OR #4 OR #5 OR #6 OR #7) *AND* DOCUMENT TYPES: (Article) 16035 |
| #14 (#8 OR #9 OR #10 OR #11 OR #12) *AND* DOCUMENT TYPES: (Article) 456779 |
| #15 ((#1 OR #2 OR #3 OR #4 OR #5 OR #6 OR #7) AND (#8 OR #9 OR #10 OR #11 OR #12)) *AND* DOCUMENT TYPES: (Article) 5342 |
| **Embase** |
| #1 adiponectin AND [1990-2017]/py 29463 |
| #2 adipq AND [1990-2017]/py 1012 |
| #3 adpq AND [1990-2017]/py 4 |
| #4 acdc AND [1990-2017]/py 109 |
| #5 gbp28 AND [1990-2017]/py 21 |
| #6 apM1 AND [1990-2017]/py 173 |
| #7 acrp30 AND [1990-2017]/py 172 |
| #8 #1 OR #2 OR #3 OR #4 OR #5 OR #6 OR #7 29779 |
| #9 'metabolic syndrome' AND [1990-2017]/py 78331 |
| #10 mets AND [1990-2017]/py 13035 |
| #11 ms AND [1990-2017]/py 440291 |
| #12 'syndrome x' AND [1990-2017]/py 66692 |
| #13 'insulin resistance syndrome' AND [1990-2017]/py 1951 |
| #14 #9 OR #10 OR #11 OR #12 OR #13 AND [1990-2017]/py 518746 |
| #15 #8 AND #14 AND [1990-2017]/py 5667 |
| **Pubmed** |
| #1 (adiponectin) AND ("1990/01/01"[Date - Publication] : "3000"[Date - Publication]) 16790 |
| #2 (ADIPOQ) AND ("1990/01/01"[Date - Publication] : "3000"[Date - Publication]) 2375 |
| #3 (ADPQ) AND ("1990/01/01"[Date - Publication] : "3000"[Date - Publication]) 2 |
| #4 (ACDC) AND ("1990/01/01"[Date - Publication] : "3000"[Date - Publication]) 71 |
| #5 (GBP-28) AND ("1990/01/01"[Date - Publication] : "3000"[Date - Publication]) 2 |
| #6 (apM1) AND ("1990/01/01"[Date - Publication] : "3000"[Date - Publication]) 121 |
| #7 (Acrp30) AND ("1990/01/01"[Date - Publication] : "3000"[Date - Publication]) 115 |
| #8 ((((((adiponectin) OR ADIPOQ) OR ADPQ) OR ACDC) OR GBP-28) OR apM1) OR Acrp30 AND ("1990/01/01"[Date - Publication] : "3000"[Date - Publication]) 17048 |
| #9 (metabolic syndrome) AND ("1990/01/01"[Date - Publication] : "3000"[Date - Publication]) 61078 |
| #10 (MetS) AND ("1990/01/01"[Date - Publication] : "3000"[Date - Publication]) 7785 |
| #11 (MS) AND ("1990/01/01"[Date - Publication] : "3000"[Date - Publication]) 278317 |
| #12 (syndrome X) AND ("1990/01/01"[Date - Publication] : "3000"[Date - Publication]) 74611 |
| #13 (insulin resistance syndrome) AND ("1990/01/01"[Date - Publication] : "3000"[Date - Publication]) 97273 |
| #14 (((((metabolic syndrome) OR MetS) OR MS) OR syndrome X) OR insulin resistance syndrome) AND ("1990/01/01"[Date - Publication] : "3000"[Date - Publication]) 448411 |
| #15 ((((((adiponectin) OR ADIPOQ) OR ADPQ) OR ACDC) OR GBP-28) OR apM1) OR Acrp30 AND (((((metabolic syndrome) OR MetS) OR MS) OR syndrome X) OR insulin resistance syndrome) AND ("1990/01/01"[Date - Publication] : "3000"[Date - Publication]) 7401 |

Table S2 Methodological quality for individual study assessed by QUADAS-2 scale

| **Study** | **RISK OF BIAS** | | | | **APPLICABILITY CONCERNS** | | |
| --- | --- | --- | --- | --- | --- | --- | --- |
|  | **PATIENT SELECTION** | **INDEX TEST** | **REFERENCE STANDARD** | **FLOW AND TIMING** | **PATIENT SELECTION** | **INDEX TEST** | **REFERENCE STANDARD** |
| Ogawa | ☺ | ☺ | ☺ | ☺ | ☺ | ☺ | ☺ |
| Gilardini | ? | ☺ | ☺ | ☺ | ☺ | ☺ | ☺ |
| Mojiminiyi | ☺ | ☺ | ☺ | ☺ | ☹ | ☺ | ☺ |
| Lee | ☺ | ☺ | ☺ | ☺ | ☺ | ☺ | ☺ |
| Boyraz | ☺ | ☺ | ☺ | ☺ | ☺ | ☺ | ☺ |
| Hata | ☺ | ☺ | ☺ | ☺ | ☺ | ☺ | ☺ |
| Patel | ? | ☹ | ☺ | ☺ | ☺ | ☺ | ☺ |

☺Low Risk ☹High Risk ? Unclear Risk

Abbreviations: QUADAS-2, Quality Assessment of Diagnostic Accuracy Studies-2.

Table S3 Quality Assessment of Included Studies on Risk Assessment

|  | Seino et al, 2009 | Nakashima  et al, 2011 | Juonala  et al, 2011 | Kim et al, 2013 | Hata et al, 2015 | Lindberg  et al, 2016 |
| --- | --- | --- | --- | --- | --- | --- |
| **Selection** |  |  |  |  |  |  |
| Representativeness of cohort with ADPQ variation | 1 | 1 | 1 | 1 | 1 | 1 |
| Selection of the cohort with normal ADPQ | 1 | 1 | 1 | 1 | 1 | 1 |
| Ascertainment of ADPQ variation | 1 | 1 | 1 | 1 | 1 | 1 |
| Demonstration that MetS was excluded or distinguished at start of study | 1 | 1 | 1 | 1 | 1 | 1 |
| **Comparison** |  |  |  |  |  |  |
| Controls for age and gender | 0 | 1 | 1 | 1 | 0 | 1 |
| Controls for BMI | 0 | 1 | 1 | 1 | 0 | 1 |
| **Outcome** |  |  |  |  |  |  |
| Assessment of MetS | 1 | 1 | 1 | 1 | 1 | 1 |
| Long enough follow-up for MetS to occur | 1 | 1 | 1 | 0 | 1 | 1 |
| Adequacy of follow up of cohorts | 1 | 1 | 1 | 1 | 0 | 1 |
| Total (9 as maximum) | 7 | 9 | 9 | 8 | 6 | 9 |

Abbreviations: ADPQ, adiponectin; BMI, body mass index; MetS, metabolic syndrome.

Table S4 Definition of metabolic syndrome adopted in enrolled studies

|  |  |  | MetS Components | | | | | |
| --- | --- | --- | --- | --- | --- | --- | --- | --- |
| Author, Publication year | Diagnostic Criteria | Definition | Obesity | Hypertrigly-ceridemia | Low-HDL-C/ high LDL-C | Hyperglycemia/  IR | Hypertension | Others |
|  |  |  |  |  |  |  |  |  |
| **Studies for Diagnostic Accuracy** |  |  |  |  |  |  |  |  |
| Ogawa, 2004 | Self-defined | At least 3 of 5 components | POW≥50%  or WC>80 cm | TG ≥120 mg/dl | HDL-C <40 or  LDL-C ≥140 mg/dl | Insulin≥15 µU/ml | SBP≥130 mmHg  (aged<10yr), SBP≥135 mmHg (aged≥10yr);  or  DBP≥80 mmHg | ALT>30IU/L |
|  |  |  |  |  |  |  |  |  |
| Gilardini,2006;  Boyraz, 2013 | Modified WHO definition for children | IR+at least 2 other components | BMI or WC > upper 97^th^ percentile value adjusted by age/sex | TG>upper 95^th^ percentile value adjusted by age/sex | HDL-C< lower 5^th^ percentile value adjusted by age/sex | HOMA-IR>median value for each Tanner stage | SBP or DBP > upper 95^th^ percentile value adjusted by age/sex | Albumin excretion rate:  (20–200 ug/min) |
|  |  |  |  |  |  |  |  |  |
| Mojiminiyi,2006 | Joint interim | At least 3 of 5 components | WC≥102cm(M)  WC≥88cm(F) | TG>150 mg/dl | HDL-C<40 mg/dl(M)  HDL-C<50 mg/dl(F) | FBG≥100mg/dl | SBP≥130mmHg, or  DBP≥85mmHg | NA |
|  |  |  |  |  |  |  |  |  |
| Lee,2007 | Modified NCEP-ATP-III for children | At least 3 of 5 components | BMI SDS≥2 | TG>upper 95^th^ percentile value adjusted by age/sex | HDL-C< lower 5^th^ percentile value adjusted by age/sex | FBG: (140-200 mg/dl) | SBP or DBP > upper 95^th^ percentile value adjusted by age/sex | NA |
|  |  |  |  |  |  |  |  |  |
| Hata, 2015;  Patel,2015 | Joint interim | At least 3 of 5 components | WC≥90cm(M)  WC≥80cm(F)  for Asians | TG≥150 mg/dl | HDL-C<40 mg/dl(M)  HDL-C<50 mg/dl(F) | FBG≥100mg/dl | SBP≥130mmHg, or  DBP≥85mmHg | NA |
|  |  |  |  |  |  |  |  |  |
| **Studies for Risk assessment** |  |  |  |  |  |  |  |  |
| Seino,2009 | Japanese criteria | Obesity+at least 2 other components | BMI>24.32 kg/m^2^ | TG≥150 mg/dl | HDL-C≤40 mg/dl | FBG≥110mg/dL | SBP≥130mmHg, or  DBP≥85mmHg | NA |
|  |  |  |  |  |  |  |  |  |
| Nakashima,2011;  Kim, 2013;  Hata, 2015 | Joint interim | At least 3 of 5 components | WC>90cm(M)  WC>80cm(F)  for Asians | TG≥150 mg/dl | HDL-C<40 mg/dL(M)  HDL-C<50 mg/dl(F) | FBG≥100mg/dl | SBP≥130mmHg, or  DBP≥85mmHg | NA |
|  |  |  |  |  |  |  |  |  |
| Juonala,2011 | Joint interim | At least 3 of 5 components | WC≥102cm(M)  WC≥88cm(F) | TG>150 mg/dl | HDL-C<40 mg/dL(M)  HDL-C<50 mg/dL(F) | FBG≥100mg/dl | SBP≥130mmHg, or  DBP≥85mmHg | NA |
|  |  |  |  |  |  |  |  |  |
| Lindberg,2016 | NCEP-ATP-III | At least 3 of 5 components | WC≥102cm(M)  WC≥88cm(F) | TG>150 mg/dl | HDL-C<40 mg/dl(M)  HDL-C<50 mg/dL(F) | FBG≥110mg/dl | SBP≥130mmHg, or  DBP≥85mmHg | NA |

Abbrevation: ALT, alanine aminotransferase; BMI, body mass index; DBP, diastolic blood pressure; F, female; FBG, fasting blood glucose; HDL-C, high density lipoprotein cholesterol; HOMA-IR, homeostasis model assessment of insulin resistance; IR, insulin resistance; LDL-C, low density lipoprotein cholesterol; M, male; MetS, metabolis syndrome; NA, not available; POW, percentage of overweight; SBP, systolic blood pressure; SDS, standard deviation score; TG, triglyceride; WC, waist circumference; yr, year.

Tabe S5. Negative predictive values and positive predictive values for adiponectin on different prevalence of Metabolic Syndrome

| MetS Prevalence (%) | NPV | PPV |
| --- | --- | --- |
| 10 | 0.97 | 0.21 |
| 20 | 0.93 | 0.38 |
| 30 | 0.88 | 0.51 |
| 40 | 0.82 | 0.62 |
| 50 | 0.76 | 0.71 |
| 60 | 0.67 | 0.79 |
| 26.6 | 0.90 | 0.47 |

NPV and PPV were calculated based on the pooled sensitivity (0.78) and speciality (0.68) in Figure 2.

Abbreviations: MetS, metabolic syndrome; NPV, negative predictive values; PPV, positive predictive value.

**Supplementary checklist**

**PRISMA checklist**

| **Section/topic** | **#** | **Checklist item** | **Reported on page #** |
| --- | --- | --- | --- |
| **TITLE** | | |  |
| Title | 1 | Identify the report as a systematic review, meta-analysis, or both. | 1 |
| **ABSTRACT** | | |  |
| Structured summary | 2 | Provide a structured summary including, as applicable: background; objectives; data sources; study eligibility criteria, participants, and interventions; study appraisal and synthesis methods; results; limitations; conclusions and implications of key findings; systematic review registration number. | 2 |
| **INTRODUCTION** | | |  |
| Rationale | 3 | Describe the rationale for the review in the context of what is already known. | 3-4 |
| Objectives | 4 | Provide an explicit statement of questions being addressed with reference to participants, interventions, comparisons, outcomes, and study design (PICOS). | 5 |
| **METHODS** | | |  |
| Protocol and registration | 5 | Indicate if a review protocol exists, if and where it can be accessed (e.g., Web address), and, if available, provide registration information including registration number. | 6 |
| Eligibility criteria | 6 | Specify study characteristics (e.g., PICOS, length of follow-up) and report characteristics (e.g., years considered, language, publication status) used as criteria for eligibility, giving rationale. | 7 |
| Information sources | 7 | Describe all information sources (e.g., databases with dates of coverage, contact with study authors to identify additional studies) in the search and date last searched. | 7-8 |
| Search | 8 | Present full electronic search strategy for at least one database, including any limits used, such that it could be repeated. | 6 |
| Study selection | 9 | State the process for selecting studies (i.e., screening, eligibility, included in systematic review, and, if applicable, included in the meta-analysis). | 6 |
| Data collection process | 10 | Describe method of data extraction from reports (e.g., piloted forms, independently, in duplicate) and any processes for obtaining and confirming data from investigators. | 7-8 |
| Data items | 11 | List and define all variables for which data were sought (e.g., PICOS, funding sources) and any assumptions and simplifications made. | 7-8 |
| Risk of bias in individual studies | 12 | Describe methods used for assessing risk of bias of individual studies (including specification of whether this was done at the study or outcome level), and how this information is to be used in any data synthesis. | 11 |
| Summary measures | 13 | State the principal summary measures (e.g., risk ratio, difference in means). | 9-10 |
| Synthesis of results | 14 | Describe the methods of handling data and combining results of studies, if done, including measures of consistency (e.g., I^2^) for each meta-analysis. | 9-11 |

Page 1 of 2

| **Section/topic** | **#** | **Checklist item** | **Reported on page #** |
| --- | --- | --- | --- |
| Risk of bias across studies | 15 | Specify any assessment of risk of bias that may affect the cumulative evidence (e.g., publication bias, selective reporting within studies). | 11 |
| Additional analyses | 16 | Describe methods of additional analyses (e.g., sensitivity or subgroup analyses, meta-regression), if done, indicating which were pre-specified. | 11 |
| **RESULTS** | | |  |
| Study selection | 17 | Give numbers of studies screened, assessed for eligibility, and included in the review, with reasons for exclusions at each stage, ideally with a flow diagram. | 12 |
| Study characteristics | 18 | For each study, present characteristics for which data were extracted (e.g., study size, PICOS, follow-up period) and provide the citations. | 13-15 |
| Risk of bias within studies | 19 | Present data on risk of bias of each study and, if available, any outcome level assessment (see item 12). | 18 |
| Results of individual studies | 20 | For all outcomes considered (benefits or harms), present, for each study: (a) simple summary data for each intervention group (b) effect estimates and confidence intervals, ideally with a forest plot. | 13-15 |
| Synthesis of results | 21 | Present results of each meta-analysis done, including confidence intervals and measures of consistency. | 15-18 |
| Risk of bias across studies | 22 | Present results of any assessment of risk of bias across studies (see Item 15). | 18 |
| Additional analysis | 23 | Give results of additional analyses, if done (e.g., sensitivity or subgroup analyses, meta-regression [see Item 16]). | 18 |
| **DISCUSSION** | | |  |
| Summary of evidence | 24 | Summarize the main findings including the strength of evidence for each main outcome; consider their relevance to key groups (e.g., healthcare providers, users, and policy makers). | 19-23 |
| Limitations | 25 | Discuss limitations at study and outcome level (e.g., risk of bias), and at review-level (e.g., incomplete retrieval of identified research, reporting bias). | 23 |
| Conclusions | 26 | Provide a general interpretation of the results in the context of other evidence, and implications for future research. | 24 |
| **FUNDING** | | |  |
| Funding | 27 | Describe sources of funding for the systematic review and other support (e.g., supply of data); role of funders for the systematic review. | 25 |

*From:*  Moher D, Liberati A, Tetzlaff J, Altman DG, The PRISMA Group (2009). Preferred Reporting Items for Systematic Reviews and Meta-Analyses: The PRISMA Statement. PLoS Med 6(7): e1000097. doi:10.1371/journal.pmed1000097

For more information, visit: **www.prisma-statement.org**.

Page 2 of 2

**QUADAS-2 checklist**

**Checklist on quality assessment of enrolled studies related to accuracy of ADPQ on MetS diagnosis based on QUADAS-2 tool**

**For Risk of Bias:**

**1. Patient selection: Could the Selection of Patients Have Introduced Bias?**

Question 1: Was a consecutive or random sample of patients enrolled? (yes for low risk, no for high risk, unclear for not reported)

Question 2: Was a case–control design avoided? (yes for low risk, no for high risk, unclear for not reported)

Question 3: Did the study avoid inappropriate exclusions? (yes for low risk, no for high risk, unclear for not reported)

**2. Index test: Could the Conduct or Interpretation of the ADPQ measurement Have Introduced Bias?**

Question 1: Were the ADPQ measurement results interpreted without knowledge of the results of the MetS definition? (yes for low risk, no for high risk, unclear for not reported)

Question 2: If a threshold was used, was it prespecified? (yes for high risk, no for low risk, unclear for not reported)

**3. Reference standard: Could the MetS definition, Its Conduct, or Its Interpretation Have Introduced Bias?**

Question 1: Is the definition likely to correctly classify the MetS condition? (yes for low risk, no for high risk, unclear for not reported)

Question 2: Were the MetS definition results interpreted without knowledge of the results of the ADPQ measurement? (yes for low risk, no for high risk, unclear for not reported)

**4. Flow and timing: Could the Patient Flow Have Introduced Bias?**

Question 1: Was ADPQ measurement performed no later than the diagnosis by MetS definition? (yes for low risk, no for high risk, unclear for not reported)

Question 2: Did all patients receive the same MetS definition? (yes for low risk, no for high risk, unclear for not reported)

**For Applicability**

**1. Patient selection:**

Are There Concerns That the Included Patients and Setting Do Not Match the Review Question? (yes for high risk, no for low risk, unclear for not reported)

**2. Index test:**

Are There Concerns That the ADPQ measurement, Its Conduct, or Its Interpretation Differ From the Review Question? (yes for low risk, no for high risk, unclear for not reported)

**3. Reference standard:**

Are There Concerns That the MetS condition as Defined by the unified definition Do Not Match the Question? (yes for low risk, no for high risk, unclear for not reported)

**NOS checklist**

**Checklist on quality assessment of enrolled studies related to risk of ADPQ variation on MetS prediction based on modified NOS scale^a^**

**Selection**

1) Representativeness of the cohort with ADPQ variation

a) truly representative of genetal adult subjects in the community **🟑**

b) somewhat representative of general adult subjects in the community **🟑**

c) selected group of subjects

d) no description on the derivation of the cohort

2) Selection of the cohort with normal ADPQ

a) drawn from the same community as the cohort with ADPQ variation**🟑**

b) drawn from a different source

c) no description of the derivation of the non exposed cohort

3) Ascertainment of ADPQ variation

a) secure record (clinical examination) **🟑**

b) written self report

c) no description

4) Demonstration that MetS was excluded or distinguished at start of study

a) yes **🟑**

b) no

**Comparability**

1) Comparability of cohorts on the basis of the study design

a) study controls for age and gender**🟑**

b) study controls for BMI**🟑**

**Outcome**

1) Assessment of MetS

a) definite MetS criteria **🟑**

b) record linkage **🟑**

c) self report

d) no description

2) Was follow-up long enough for MetS to occur

a) yes (if follow up period>3 years) **🟑**

b) no

3) Adequacy of follow up of cohorts

a) complete follow up for all subjects **🟑**

b) cohorts with follow up rate≥80% **🟑**

c) cohorts with follow up rate < 80%

d) no statement

^a^ Study can be awarded a maximum of one star for each numbered item within the Selection and Outcome categories. A maximum of two stars can be given for Comparability item.
